# Theory and Validation of Magnetic Resonance Fluid Motion Estimation Using Intensity Flow Data

**DOI:** 10.1371/journal.pone.0004747

**Published:** 2009-03-09

**Authors:** Kelvin Kian Loong Wong, Richard Malcolm Kelso, Stephen Grant Worthley, Prashanthan Sanders, Jagannath Mazumdar, Derek Abbott

**Affiliations:** 1 Centre for Biomedical Engineering and School of Electrical & Electronic Engineering, University of Adelaide, Adelaide, South Australia, Australia; 2 School of Mechanical Engineering, University of Adelaide, Adelaide, South Australia, Australia; 3 School of Medicine, University of Adelaide, Adelaide, South Australia, Australia; University of Hull, United Kingdom

## Abstract

**Background:**

Motion tracking based on spatial-temporal radio-frequency signals from the pixel representation of magnetic resonance (MR) imaging of a non-stationary fluid is able to provide two dimensional vector field maps. This supports the underlying fundamentals of magnetic resonance fluid motion estimation and generates a new methodology for flow measurement that is based on registration of nuclear signals from moving hydrogen nuclei in fluid. However, there is a need to validate the computational aspect of the approach by using velocity flow field data that we will assume as the true reference information or ground truth.

**Methodology/Principal Findings:**

In this study, we create flow vectors based on an ideal analytical vortex, and generate artificial signal-motion image data to verify our computational approach. The analytical and computed flow fields are compared to provide an error estimate of our methodology. The comparison shows that the fluid motion estimation approach using simulated MR data is accurate and robust enough for flow field mapping. To verify our methodology, we have tested the computational configuration on magnetic resonance images of cardiac blood and proved that the theory of magnetic resonance fluid motion estimation can be applicable practically.

**Conclusions/Significance:**

The results of this work will allow us to progress further in the investigation of fluid motion prediction based on imaging modalities that do not require velocity encoding. This article describes a novel theory of motion estimation based on magnetic resonating blood, which may be directly applied to cardiac flow imaging.

## Introduction

Medical imaging techniques such as velocity-encoded (VENC) phase contrast magnetic resonance imaging [1], [2], [3] is able to produce flow measurements of blood in heart structures and enables a clear evaluation of the cardiac functions. However, phase contrast magnetic resonance image scans take a longer duration than non velocity-encoded imaging protocols such as True FISP (Fast Imaging in Steady State Free Precession) [4], [5], [6]. This creates a need to have a magnetic resonance imaging velocimetry methodology, which requires less processing time and is unaffected by the absence of the velocity-encoding protocol.

Multi-resolution motion estimation [7] on intensity magnetic resonance (MR) images is able to predict fluid motion information, within signal images, without direct and physical measurement of the fluid velocity field. Different degrees of turbulence in fast flowing blood as well as inconsistency in magnetic resonance due to flow from different directions produce de-phasing of proton spins at different levels and results in intensity contrast of the image pixels [8], [9]. We propose a methodology for computationally determining the movement of fluid in a vessel based on motion estimation of the contrasting MR-signals, instead of encoding velocity information onto flow images during scanning. However, such medical scanning modalities cannot be applied to any fluid to enable flow field generation. The lack of hydrogen nuclei in most flow media such as air results in void signal registration. On the contrary, it works relatively well on magnetic resonating blood flow.

This paper explains the theory of magnetic resonance fluid motion estimation in detail. It includes a study to evaluate the MR fluid motion estimation technique by matching a computationally predicted flow with an analytically determined one. In our experiments, we assume the analytical information to be the true reference (i.e. ground truth) data for comparison. Flow vector differencing in the Cartesian and radial grid can be carried out to determine the error of the motion estimation and assess the accuracy of the computational algorithm used in the motion tracking system.

The verification process indicates that extension of motion estimation onto magnetic resonance images of dynamic fluids, such as blood within the heart chamber, within this prototypical framework can be carried out. The estimation of blood flow field within the human heart chamber can be performed to give an indication of the flow behavior and can be used to investigate cardiac functions.

We examine some of the well-established velocimetry systems that exist to generate accurate vector fields of fluid flow of one temporal and up to three spatial dimensions. It is interesting to highlight that such flow tracking can be classified as either optical-, magnetic resonance- and ultrasonic- image velocimetry. More importantly, we are interested to look at in-plane flow field generation using the different types of imaging systems. Particle image velocimetry (PIV) can serve as a validation tool for verifying magnetic resonance imaging velocimetry (MRIV). For example, two dimensional phase contrast flow measurement [10] has been verified against experimental methods using PIV [11] previously. On the other hand, phase contrast magnetic resonance imaging of cardiac flow has been compared against that of ultrasonic imaging in various studies [12], [13].

### A. Optical-based Image Velocimetry

Over the past few decades, particle image velocimetry [14], [15] has been an established method of performing flow tracking based on optical scanning of fluid suspended particles. Nano-particles in the form of glass beads can act as velocity trackers when illuminated by a two-dimensional sheet of high-powered laser source. Cross-correlation of concomitant images [16] is performed based on snap shots of the flow scenario at two instances dictated by a measurement interval that is dependent on the speed of the flow. The computation of collective particle displacements gives a two-dimensional flow visualization of the fluid. However, the limitation of PIV in imaging flow through non-compatible optic structures has been an obstacle in studying cardiovascular flow in the human body.

Although the particle image velocimetry system utilizes the cross-correlation of particle windows in the optically captured image to compute the localized direction of flow, the motion estimation scheme can also be used for such optical-based experimental flow studies [17], [18], [19]. Parametric flow fields generated by the optical flow algorithms used in these studies compared favorably with results obtained from the well-established computational component used in particle image velocimetry.

### B. Magnetic Resonance-based Image Velocimetry

The magnetic resonance-based image velocimetry is VENC phase contrast MR imaging, which encodes velocity information onto images in real-time during scanning. In contrast to predicting fluid motion based on illuminated nano-track particles in optically compatible vessels, this approach extracts MR signals produced by nuclear spins within a fluid, determines the phase shift of the transverse magnetization during movement of the spin ensemble from the stationary spins, and encodes this information as pixel intensity onto an image [10], [20]. Since the phase shift is directly proportional to the velocity of the fluid, deciphering velocity components from the intensity images pertaining to the in-plane horizontal and vertical orientations, and reconstructing them, provides motion fields of up to one vector per pixel. For MR image-based velocimetry, we are able to image through the heart, whereas PIV is optical-based, and therefore flow imaging within opaque cardiac structures is impossible.

### C. Ultrasonic-based Image Velocimetry

Doppler ultrasound, as its name implies, is based on Doppler shift caused by blood scatter movement and is a widely accepted technique for visualization of blood flow patterns. Analysis of the flow field obtained by ultrasound methods enables useful results in cardiac diagnosis [21]. However, the Doppler ultrasound output is usually represented as a two dimensional image.

Medical ultrasound works by generating high frequency electrical pulses and using piezoelectric elements of a transducer to convert them into mechanical vibrations. The emission of ultra-frequency sound and detection of sound waves from the resulting echoes is performed by transducers. After conversion into electrical signals, processing is carried out to decipher blood flow velocities [22].

Real-time blood motion imaging using color sonograms can be utilized. For this medical imaging modality, the speckle pattern from the blood flow signal is preserved, enhanced, and visualized [23], [24]. In this technique, a high frame rate is necessary for acquiring speckle pattern motion due to the rapid decorrelation of the speckle pattern from blood flow. In addition, good spatial resolution of the speckle pattern is essential.

A huge limitation of ultrasound imaging is that the Doppler shift is only sensitive to the velocity component in the orientation of the ultrasonic beam. However, clinical examination can be achieved with low cost and produces real-time flow visualization. In addition, ultrasound systems can be highly portable now [25]. This makes Doppler sonography more clinically attractive to use than magnetic resonance imaging. Despite these system advantages, a flow projection onto a plane for an accurate slice assessment of the cardiac flow is difficult to perform. Based on this aspect, it is inferior to velocity-coded MR imaging that can reconstruct accurate temporal flow grids of up to three spatial dimensions [26].

## Materials and Methods

### A. Theory

#### A.1. True FISP Magnetic Resonance Imaging

We examine nuclear magnetic resonance (NMR) imaging at a basic level to aid clarity of the quantum mechanical concepts. An organic structure positioned within the centre of an external magnetic field becomes itself partially magnetized with a magnitude of comparatively lower order. The assemblage of hydrogen nuclei (protons) in the water molecules within the body can be perturbed with radiofrequency radiation. The nuclear spins then realign with the magnetic field and emit radiofrequency (RF) waves during this longitudinal relaxation period [27]. Time 1 (*T_1_*) is defined as the duration for nuclear realignment and emission of RF signals that can be registered onto the MR images that we use for examination. Since the rate of emission of the RF waves is dependent on the type of material that contains the nuclei, different intensity of pixels representing the tissues can distinguish various anatomical structures.

Based on a similar type of magnetic field configuration, pulses of radio waves that have their magnetic moments perpendicular to the magnetic field applied can cause the hydrogen nuclei to have magnetic moment transverse to their original orientation. Realignment of nuclear spins after this transverse magnetization has a decay time constant labeled as Time 2 (*T_2_*) for this transverse relaxation. Likewise, for tissue classification, the rate of decay is dependent upon the material nuclei, and therefore registers differently onto the MR image that is made up of pixels with varying intensity.

True FISP MR imaging is a modality capable of imaging cross-sections of cardiac structures with unsurpassed soft tissue contrast [28]. It is one of the most popular medical imaging modality for registration of physiological properties of the heart and arteries. True FISP MR imaging combines both longitudinal and transverse magnetization [29]. It is characterized by a complex *T_2_*/*T_1_*-contrast configuration [30] and refocuses all gradients over a repetition interval, thereby achieving fast imaging with high signal [29].

#### A.2. Asynchronous Precession of Hydrogen Nuclei in Turbulent Flow

This section describes the theory governing the concept of void signal registration due to turbulence within fluid whose atomic nuclei have been aligned either parallel or anti-parallel to a powerful and uniform magnetic field. As the high-energy nuclei relax and realign, they emit energy with certain properties that are recorded to provide information about the medium. Image contrast is created by weighting the energy signal during realignment of the nuclear spins with the magnetic field.

A signal image is generated as a result of this quantum mechanical activity. As the fluid is transported, the same signal from nuclei that is retained within the fluid follows the displacement. In a turbulent flow, the diffusion of magnetic moments occurs [31]. Protons in the hydrogen nuclei of molecules can be given a phase spin and the de-phasing (asynchronous precession) of spin due to turbulence in flow gives a low MR signal during imaging [8], [9]. Therefore, there is a reduction in the signal registration onto the image. Effectively, diffusivity of spin protons at a point is represented by a reduction in signal intensity in the image [31]. Because the diffusivity follows the movement of the fluid in a channel, there exists an intensity change in the direction of flow. The intensity contrast of the diffusion in the image becomes greater as the speed of the fluid flow increases.

#### A.3. Non-stationary Patterns of Varying Intensity in Cine-MR Imaging

In the previous section, we discuss the non-uniform and temporal intensity of nuclear signal registration of chaotic flow due to de-phasing of the proton spins. We also discuss the nuclear characteristics of blood within the human heart that is quantum excited under the magnetic resonance scheme. It must also be emphasised that in the heart chambers, the nuclear spins may move perpendicularly in and out of the imaging plane. Therefore, spins that receive the original excitation may, in turbulent flow, not experience magnetic resonance gradient refocussing. Likewise, spins that do not receive the original excitation may in fact move into the imaging plane after the RF excitation pulse, and since they are not quantum mechanically stimulated to begin with, no MR-signal may be returned. Extrapolating this concept further, some signal loss due to fast flowing jets are probably due to spins moving too quickly to be excited and refocussed, creating signal voids [32].

Due to the inhomogeneous presence of asynchronous proton spins, nuclear signals emitted from dynamic fluid displays on MR images as varying patterns of intensity depicting the blood flow movement. It may be worthwhile mentioning that poor temporal and spatial resolution imaging may blur the observation of blood movement between consecutive images. At some phases of scans, the presence of low-turbulent regions may also weaken the intensity contrast variation in blood images, so that visual tracking of blood motion declines in accuracy.

#### A.4. Motion Estimation of MR-signals

A series of MR images are presented in cine-mode as the fluid is in motion. The velocity of dynamic fluid is quantified in real time by numerically computing the shift of intensities within the quantized regions of each set of temporally consecutive images. A velocity flow field can be constructed using a graphical plot and other fluid dynamics properties can be derived from the velocity flow measurement. From the results, the characteristics of the fluid flow can be analyzed using these properties.

We have developed a methodology for computationally determining the movement of fluid in a vessel based on motion estimation of the contrasting MR-signals. The motion of localized turbulence is influenced by the general flow globally. Motion estimation using multi-resolution optical flow technique is able to track the movement of the flow at various resolutions and resolve them to produce a global flow field in two dimensions. Therefore, we term this approach as MR fluid motion estimation, as it is able to compute the motion of MR imaged fluid.

**Figure 1:**
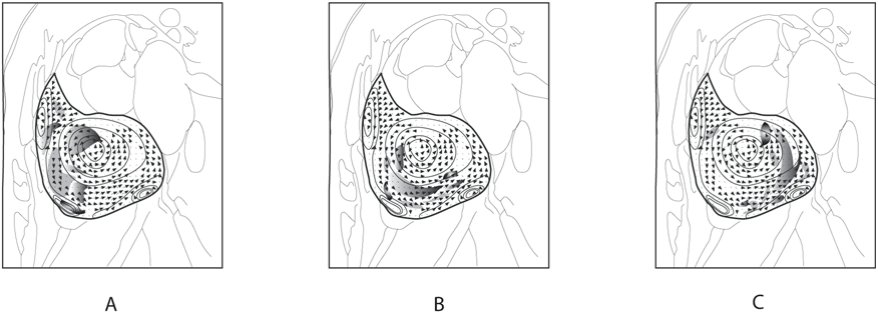
Motion estimation of in-plane MR-signals. Based on schematic display of a right atrial flow, the ensembles of asynchronous proton spins that show up as contrasting signal intensity on the cine-magnetic resonance images are represented by grey patches of varying intensity. Using a fluid motion estimation scheme, velocity vector fields pertaining to the blood flow images of arbitrary (*n*−1), *n* and (*n*+1) phases in a cardiac cycle of *N* phases can be predicted.

Application of flow based on the use of motion estimation algorithm allows us to produce flow vectors over the region of analysis defined. The technique makes use of images from two subsequent phases to predict the flow field. Typically, cine MR image scanning results in a sequence of *N* phases. Post-processing of the data from (*N*−1) pairs of images gives a series of flow field displays for evaluation and analysis. As such, predicting the ensemble movement of asynchronous proton spins represented in magnetic resonance images of a heart chamber can be technically feasible (Figure 1).

#### A.5. Computational MR Fluid Motion Estimation

**Figure 2:**
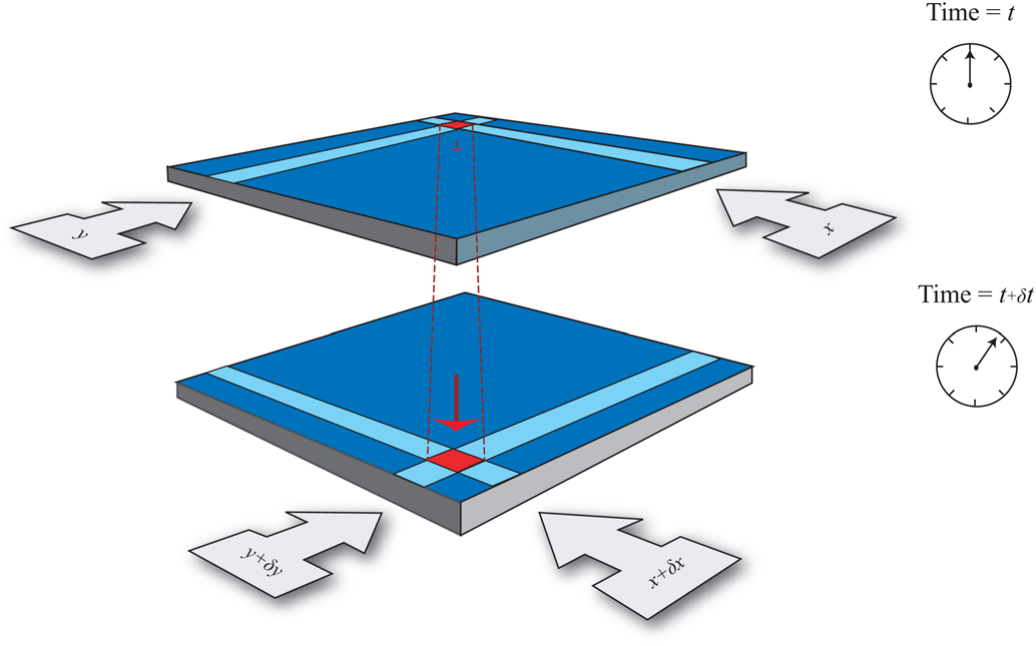
Estimating spatial motion of pixel using optical flow. Assume the shift of pixel from position (*x*, *y*, *t*) to (*x*+*δx*, *y*+*δy*, *t*+*δt*). The derivatives of *x* and *y* with respect to *t* gives the *x* and *y* components of the spatio-temporal signal flow respectively. The optical flow motion constraint allows us to derive these velocities up to one vector per pixel.

A method of performing fluid motion tracking using True FISP MR images of non-stationary flow has been suggested recently. In our approach, the optical flow algorithm which belongs to a class of motion estimation [33] is utilized. It generates flow vectors that correspond to the apparent motion of brightness or intensity patterns in the image (Figure 2). We have described prediction of the intensity flow displacement based on optical flow constraint mentioned in Appendix S1.

**Figure 3:**
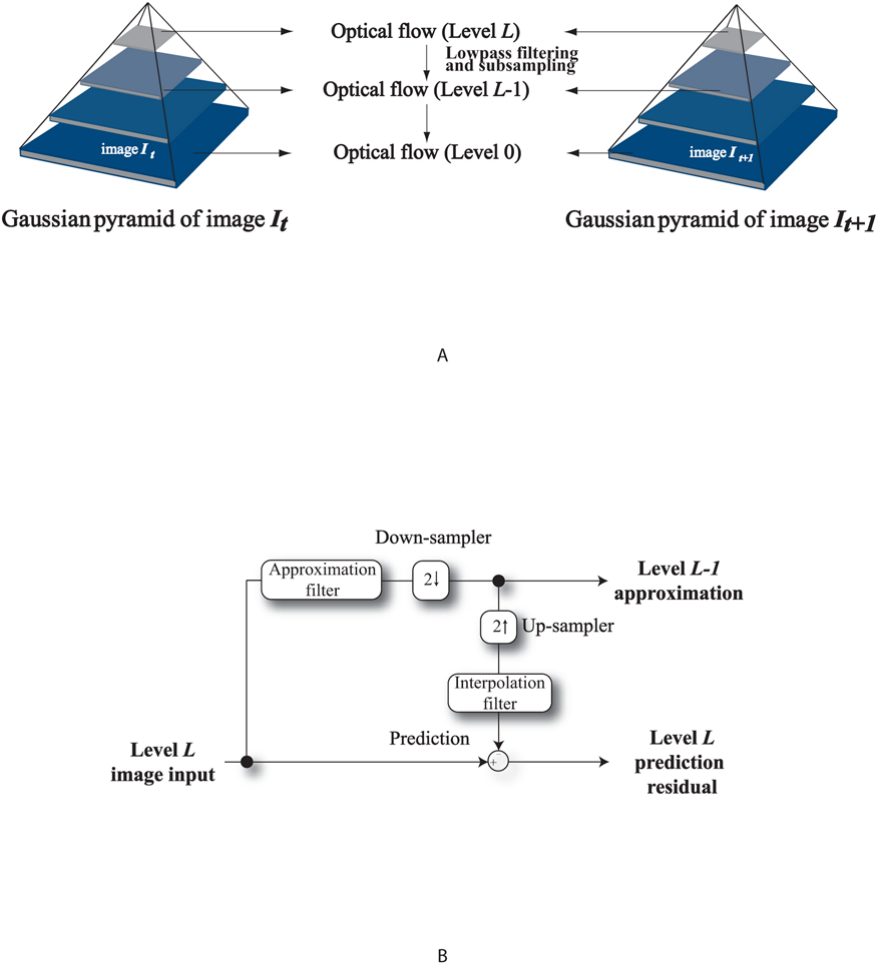
Multi-resolution motion estimation using pyramid implementation. Diagrammatic view of the Gaussian pyramid with optical flow applied onto every image level (0 to *L*) is presented in (a). Each level in the pyramid is a sub-sampled version of the level below. In the first step, the optical flow between the top level images is computed. We project the computed coarse-level flow field onto the next finer pyramid level and continue this at each level of the pyramid until the finest pyramid level has been reached. The system block diagram in (b) gives an illustration of the algorithmic operation of this pyramidal implementation.

In our optical flow scheme, the pyramidal Lucas Kanade optical flow method [7], which incorporates a multi-scale approach, has been applied to support large scale fluid motion and for improved accuracy. A top-down estimation of the flow by using an image pyramid is performed, with the apex representing the MR image at a coarse scale. Computational results from this level are passed to the next and this process is carried on based on the flow estimated at the preceding scale until the original scale is reached. We refer to the diagram in Figure 3 to illustrate the computational aspect of pyramidal optical flow. Projection of the computed coarse-level flow field onto the next finer pyramid level is continued for each level of the pyramid until the finest pyramid level has been reached.

For signal emitting nuclei motion that are represented by intensity pixels on MR images, the application of multi-resolution optical flow scheme that predicts fluid motion is based on grey-level constancy assumption or the optical flow constraint [33]. The accuracy of motion estimation critically depends on the magnitude of image motion. In fact, depending on the spatial image frequency, very large motions even may cause aliasing along the time frequency axis. For a fixed global velocity, spatial frequencies moving more than half of their period per frame cause temporal aliasing [17]. Therefore, a suitable temporal resolution of the imaging is required for accurate tracking.

### B. Generation of Test Data

The validation of a proposed or an implemented system can be achieved if analytical data can be created to calibrate its performance deviation from the perfect situation. We examine its characteristics and make necessary improvements to the configuration. This section presents the equations for describing analytical flow field that will be used in our system performance calibration.

#### B.1. Analytical Formulation of Vortex

The Oseen vortex [34], [35], [36] has analytically defined velocity, vorticity and circulation. If Γ_0_ is the circulation, and *L* is the length scale corresponding to one standard deviation of the Gaussian vorticity distribution of the vortex, we can define the angular velocity *ω*(*r*) in Eq. 1 as
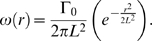
(1)


We express the tangential velocity *V_θ_*(*r*) as a function of *r* in Eq. 2 such that
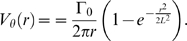
(2)


**Figure 4:**
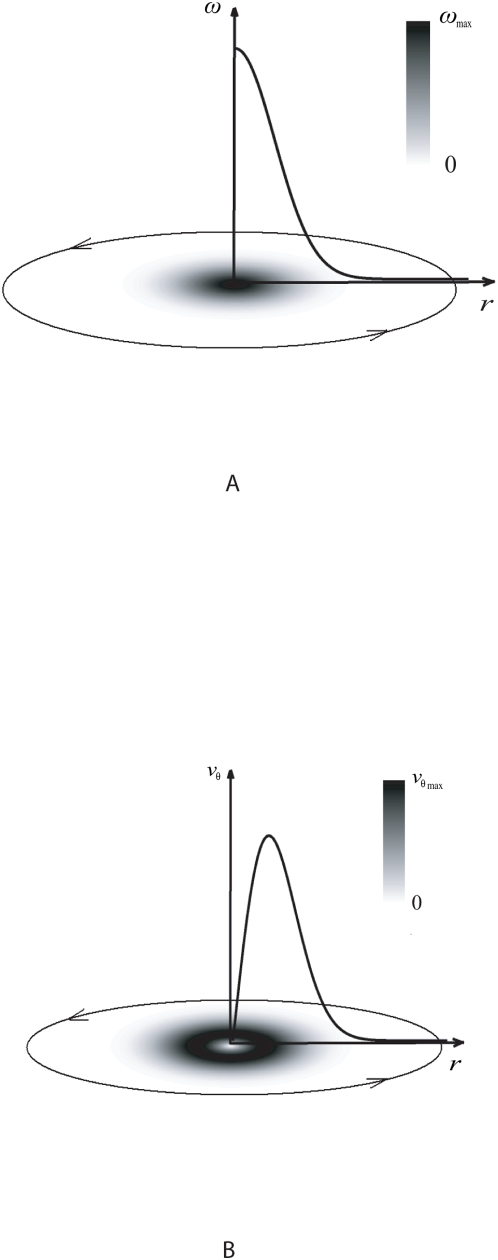
Velocity Characteristics of Oseen Vortex. Variation of profiles with respect to vortex radius *r* is shown for the angular and tangential velocities labelled as *ω* and *v_θ_* in (a) and (b) respectively. The profile of magnitudes from the core to boundary of vortex can be represented using varying gray-scale intensity. We have computed the variation of the presented vortex based on analytical formulations.

The parameters for our data generation are set as Γ_0_ = 1 mm^2^/s, and *L* = 1 mm. The computational domain range is −20*L*≤*r*≤20*L*. We can digitize the analytic velocity field over a Cartesian grid with each coordinate denoted by (*x*,*y*), and with velocity interrogation spacing, *δ* to produce a velocity vector flow field, *u_x,y_* at a resolution of *δ*/*L*. This allows us to quantify the effect of velocity field resolution based on the analytical data produced for our computational approach. The velocity profiles generated by these equations are plotted as a function of *r* (Figure 4). The angular and tangential velocities vary with respect to the radius of the vortex and their magnitudes can be represented using gray-scale intensity. Note that the tangential velocity at the core is zero despite having a finite vorticity. The velocities *ω* and *v_θ_* vary from 0 to maximum values of *ω*_max_ and *v_θ_*_max_ respectively.

#### B.2. Generating Vortex Tracks for Artificial Data

We map the analytical velocity field onto a rotational grid to generate discrete vortical tracks with radial intervals in polar coordinates. We shade alternatively spaced intervals and use the gray-scale intensity based image configuration as test data for the fluid motion estimation technique. The spatial dimensions of the track intervals can be varied to quantify the error due to decrement of feature quality.

**Figure 5:**
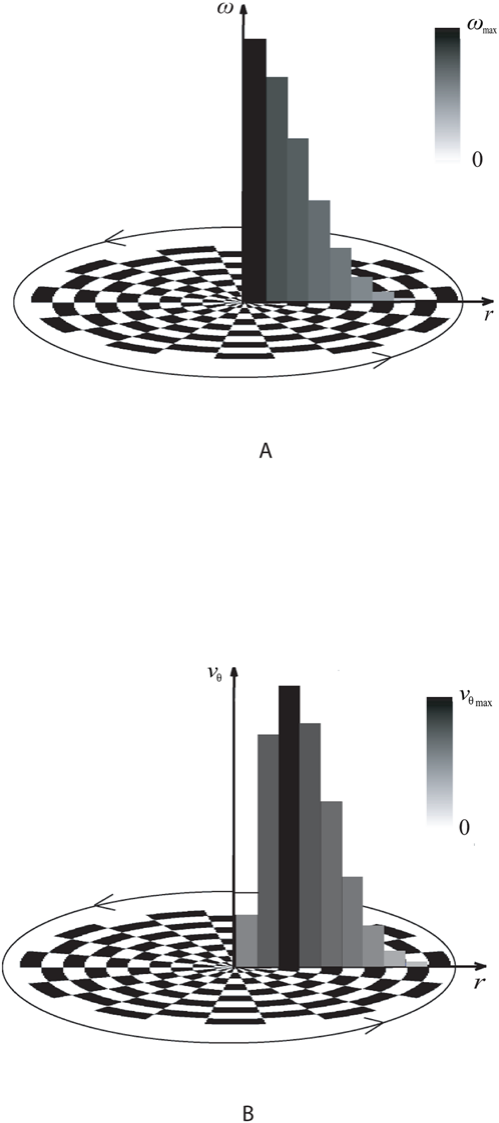
Artificial flow grid based on Oseen vortex formulation. Gray-scale intensity based polar grid with alternating contrast track intervals in a rotational fashion representing the vortical tracks of motion is demonstrated with (a) and (b) describing the variation of angular and tangential velocities respectively. The velocity variation is discrete based on the configuration of the grid, which is constructed using alternating dark and bright segments in the radial and angular directions.

The contrasting intensity for alternating track intervals provides track features for the motion estimation algorithm in this experiment. The motion of the grid is then represented using a series of these intensity based images that display the change in positions of the segments according to each track angular velocity to give an optical effect of the rotation. This causes track rings corresponding to specific radial locations to rotate at different speeds uniformly and according to the profile of the angular velocity in Figure 5. Such a configuration gives a velocity profile that has discrete values. It then is possible to quantify this rotation from the optical perspective using established motion-tracking algorithms.

#### B.3. Fluid Motion Estimation Flow Prediction

An applied computer algorithm [37] enables a multi-resolution Lucas Kanade feature tracker. Flow map at the pixel resolution; wherein, a dense velocity field (one velocity vector per pixel) is generated. We also implement removal of outliers which we classify as high magnitude flow vectors. Replacement of void flow vectors from this removal is performed by means of image field synthesis from neighborhood flow maps which we will describe in the next section. Then, calculated vectors are scaled to match the analytical vector set.

#### B.4. Filtration of Flow Outliers and Vector Synthesis

We sought to devise a way of removing outliers in the flow vector field. Optical flow vectors that pertain to very large pixel displacements relative to that of the pixels in its adjacent region are isolated using the median test [15]. We perform clustering of optical flow vectors with magnitudes above a user defined threshold. The cluster of flow vectors is classified as outliers provided that two conditions are fulfilled: (1) the difference between the vector magnitude *U* and the median magnitude *U_m_* in the flow field of size (*X*,*Y*) exceeds a threshold denoted by *τ*_mag_ which can be arbitrary set, and (2) the number of vector items within the group that is encapsulated by a sampling window size (*w_x_*,*w_y_*) which met the first condition do not fall below a specific value *τ_n_*. The two conditions that qualify the vectors in the flow field as outliers are stated mathematically as follows:
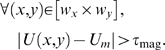
(3)

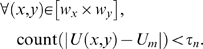
(4)


Voids due to removal of these outliers must be filled in with new vectors for the flow to be continuous. We devised a simple approach of growing vectors using a flow field synthesising approach. This technique is analogous to the occlusion fill-in algorithm used in texture synthesis by non-parametric sampling [38] except that the sampled elements are flow vectors instead of texels (texture elements). We also can apply reduction in resolution to the flow field. The averaging of vectors within interrogation windows results in a lower resolution of flow field but also can help in smoothing of flow data by negating the effect of high magnitude outliers with more accurate vectors in its neighborhood regions.

#### B.5. Variation of Vortical Track Interval Size

We vary the resolution of track intervals in the vortex to investigate flow tracking accuracy of fluid motion estimation. This may be achieved by varying the spatial density of contrasting grid intervals pertaining to vortical track items of the intensity image in the polar directions. We define the count of track intervals within the entire polar grid to be *υ* so that
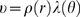
(5)and *ρ*(*r*) and *λ*(*θ*) denote the number of tracks and radial sections respectively at each configuration of the vortical tracks.

#### B.6. Configuration of Tracking Features

The configuration of the intensity based polar grid will affect the feature quality used in our tracking experiments. This has to coincide with the settings of the motion estimation algorithm. We design three parameters for conducting our error estimates of the computational approach. The algorithm sampling size *W* is varied with increments at 1 pixel from 2 to 20 pixels along with the same number of increments of image size, *I* at 10 pixels from 160 to 320 pixels. We have specified the number of tracks *ρ* and radial sections *λ* for levels 1 to 3 as tabulated in Table 1.

**Table 1:** Configuration characteristic of gray-scale track grid.

level	*ρ*	*λ*	*υ*
1	20	10	200
2	20	22	440
3	20	30	600

level -Identification number of configuration.

ρ -Number of tracks.

λ -Number of vortical track intervals.

ν -Total number of track intervals.

Variation of ρ and λ will adjust the density of the signal features used in motion tracking. The number of track intervals is an indication of the resolution of features in the track grid represented as an image.

A higher value of *λ* or more sectioning of the polar grid into tracks at angular intervals gives a smaller track-interval size, and generates finer features that are densely located near the core of rotation. This corresponds to more intensity based track features but at the expense of quality reduction, which has an effect on tracking accuracy of the fluid motion estimation. Adjustment to the optical flow algorithm, such as the sampling window size, can be carried out to improve tracking.

#### B.7. Variation of Image Size and Optical Flow Window Size

We demonstrate the effect of the test image size, denoted by *I*, and also the pyramidal window size, *W* on the accuracy of the optical flow algorithm. We increase the resolution of the track outlines using increments of image size. Therefore, a larger image size will result in a higher resolution image and quality of features used for tracking. The improvement in quality of signals will have an effect on tracking, and increasing the size of the sampling window, which is the interrogation mask used in the tracking, will capture a larger quantity of the signals but at the expense of overly smoothing the velocity flow field due to the larger relative size of the sampling window with respect to the image.

#### B.8. Variation of Noise Addition and Smoothing Filter Mask Size

In a separate study, we used a standard test intensity image. We applied multiplicative Gaussian noise to it at various percentages, followed by smoothing using a filter based on pixels averaging within a mask of size *n*. The addition of noise to the images will cause the tracking to lose accuracy; however, smoothing of the images subsequently suppresses noise and reduces the error. Nevertheless, there is still a specific threshold to the addition of noise such that this suppression is able to still maintain accurate tracking.

### C. Methods of Computational Data Validation

Our objective is to compare the velocity field estimate with that of the analytical one based on the direction and magnitude of the vectors represented using a flow image whereby every pixel stores each of the velocity in the *x* and *y* directions. Note that the spatial resolutions of analytical and computational flow images have to be the same for flow image differencing to take place.

#### C.1. Magnitude of Velocity Vectors in Radial Direction

For a track grid of radius *R*, the average magnitude of tangential vectors (from various angles) obtained from the track location that is extended by a circumference of radius *r*, is compared with the analytical velocity magnitude based on the same radius of *r*. For each track at that radius, the computational velocity is computed by taking the average of *N_θ_* number of tangential velocity vectors arranged circumferentially about the centre of rotation. This procedure is performed based on *N_r_* numbers of *r* variables from the vortex centre to edge of the computational domain up to *R*. Note that 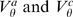 represent the analytical and computational tangential velocity respectively. Error based on the difference between the computational velocity and the analytical velocity at the defined polar coordinates can be generated using
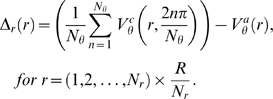
(6)


A graph of 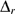 versus *r* can be produced for detecting regions of unacceptable errors due to image signal aliasing, which usually results from poor definition of closely packed track features.

#### C.2. Magnitude of Velocity Vectors in Cartesian Grid

The average magnitude of tangential vectors is compared with the analytical velocity magnitude at every coordinate (*x*, *y*) where (*I_x_*, *I_x_*) is the size of the flow field in the *x* and *y* directions. Note that (*u_a_*, *v_a_*) and (*u_c_*, *v_c_*) represent the velocity at *x* and *y* directions pertaining to the analytical and computational velocity image grid respectively. The error function based on Δ*_v_*(%) for *u* and *v* velocity components at every *x* and *y* coordinates is given by
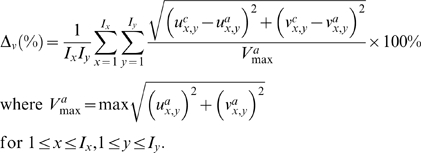
(7)


The system response that is based on Δ*_θ_*(%) can be plotted. This error value will be high if sampling window sizes that falls below a specific threshold value. The mismatch of interrogation sampling window sizes with respect to image size will result in sub-optimal tracking accuracy.

#### C.3. Direction of Velocity Vectors in Cartesian Grid

We take the angular difference in the sets of flow vectors produced using two different systems by performing the following mathematical operation to produce the average of all absolute angular vector differences in percentage, Δ*_θ_*(%) where *θ^a^* and *θ^c^* represent the orientation of vectors for analytical and computational flow fields respectively. This means the difference between the computed and the Oseen vortex velocity values such that
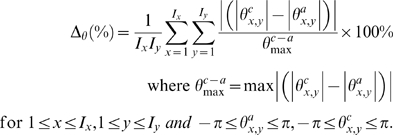
(8)


A graph based on this error function for variation of image size, *I* and sampling window size, *W* can be plotted to demonstrate the effect of improvement in feature quality that follows the increment in image size, and the required proportional increase in sampling window for interrogation of the features.

## Results

Here, we describe the results of velocity field estimates based on variation of the various parameters described in the previous sections. The aim of this study is to evaluate the performance of fluid motion tracking in terms of computational accuracy.

### A. Velocity in Image Representation

Error analysis is carried out to give an indication of the angular difference of the analytical and computational results. We prepared the error surface response curve that depicts the influence of image and sampling window sizes on the discrepancies in the flow vectors for analytical and computational flow results.

**Figure 6:**
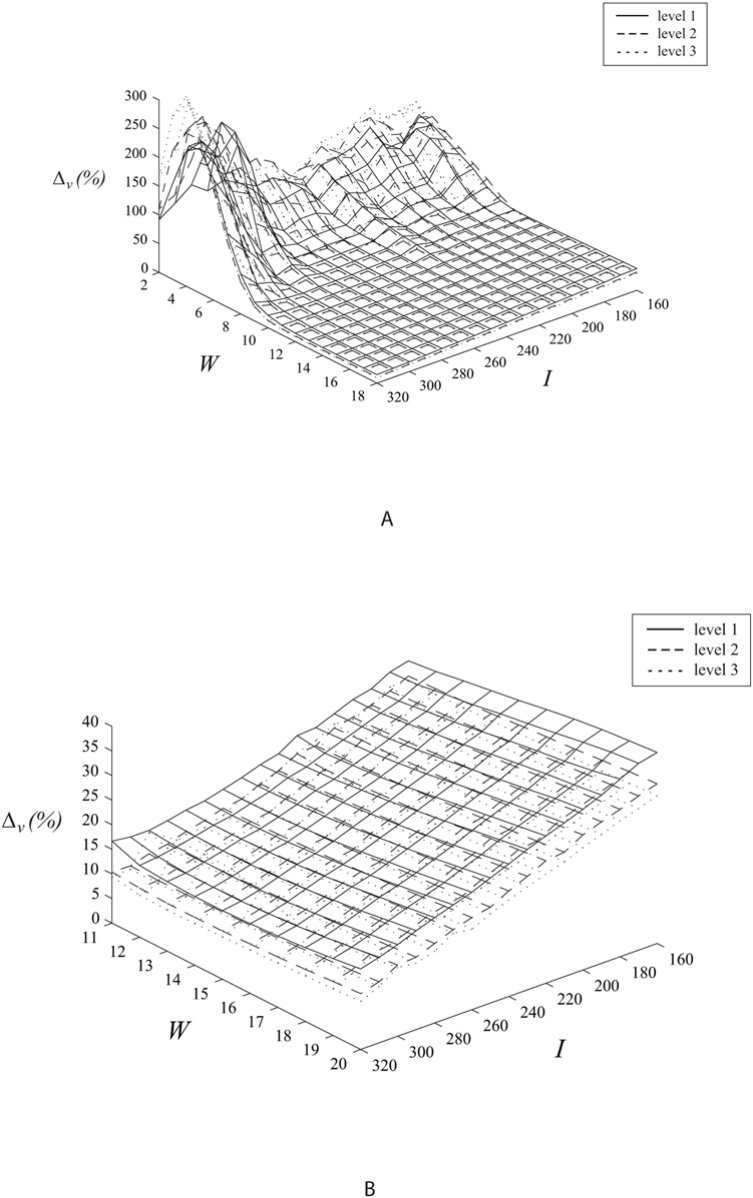
Tracking accuracy based on tangential velocities. Flow vector velocity differences based on variation of sampling window size versus image size with different levels of track interval sizes are demonstrated. In this experiment, the sampling window and raw image size dimensions are varied to analyse the tracking effect of the system. The results demonstrate that tracking improves when the image size increases which can be accredited to the increase in resolution and corresponding upgrade of the quality of track features.

High fluctuation of error estimates exist due to small sampling window sizes with dimensions of 2 to 10 pixels (Figure 6). Such sampling configurations are undesirable for the motion tracking algorithm. The increment of sizes from sampling window widths of 11 pixels onwards shows relatively small variation in the tracking accuracy.

**Figure 7:**
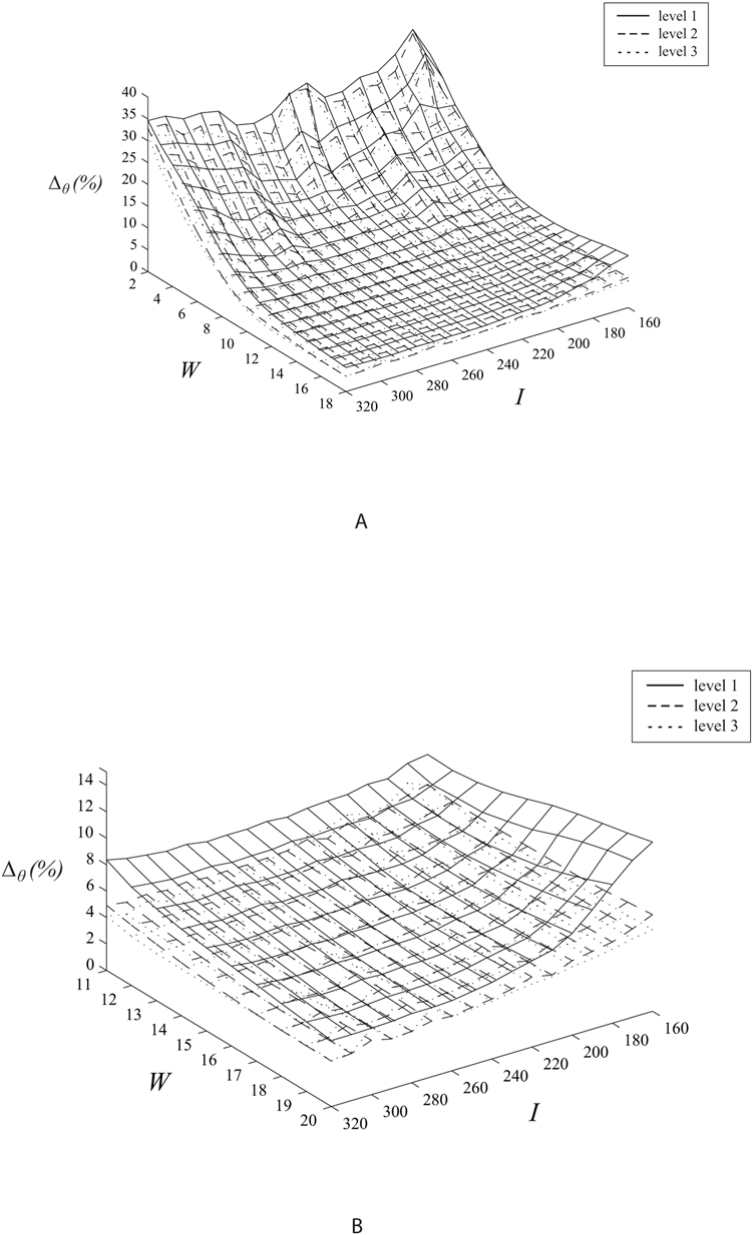
Tracking accuracy based on angular velocities. Flow vector angular differences are shown based on variations of sampling window size versus image size with different levels of track interval dimensions (i.e. levels 1 to 3). Similar to the results shown for variation of angular velocity, this set of graphs demonstrates that the tracking is more stable for sampling window sizes from a recommendation of 11 pixels onwards. Angular velocity error decreases as the image becomes dimensionally bigger.

Larger image sizes give a reduction in error estimates and can be further reduced by a corresponding increment in the sampling window size used by the algorithm for image sizes from 260 by 260 pixels onwards (Figure 7). The improvement in tracking accuracy due to increase in image size can be explained by the enhancement of feature quality due to the larger number of pixels used to represent varying space of high and low intensity track intervals. In addition to this observation and explanation, we can further deduce that an increase of sampling window size at the right image proportion will improve tracking accuracy due to capture of the required features at appropriate interrogation space.

Image and sampling window sizes at the lower and upper limits of *I* and *W* dimensions respectively gives a high error. Note that *I* and *W* at the upper and lower limits also give a relatively high error. Both *I* and *W* at the upper limits of their dimensions respectively give the smallest error. There is a reduction in error following an increase in image size. However, for large images, coupling with a proportionally incremented sampling window size used in motion estimation can further reduce error.

### B. Velocity in Radial Direction

We observed the deviation of velocities in the radial direction for both computational and analytical values based on variation resolution, noise and smoothing of track features.

#### B.1. Variation of Track Feature Resolution

Motion estimation is performed using a sampling window size of 20 by 20 pixels for a 260 by 260 pixel image. These dimensions are arbitrarily chosen such that the size of the image is 13 times that of the sampling window. The image is a spatial representation of the circular track grid based on three configurations (labeled as levels 1 to 3). A series of images can provide temporal representation of the intensity grid that is rotating. The image and sampling configurations have been set in such a way that the computational profile of the velocity tracking clearly deviates from the analytical one and can provide a good illustration of how the track density can affect the motion estimation algorithm. Therefore, it is irrelevant to set high image resolutions for achieving accurate motion tracking here.

We have sampled the tangential velocity values in the radial directions for *N_θ_* at 10 counts from 0 to 360 degrees at an interval of 36 degrees circumferentially about the centre of rotation. The counts *N_r_* is taken to be 50 samples from *r* = 0 to 5 mm at intervals of 0.1 mm. Note that velocities *V_θ_* and 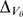 both have units of mms^−1^. For a standard comparison, we scale the maximum peak velocity to be 10 mms^−1^.

Our analysis is based on 10 samples taken in the angular direction. The accuracy of profile of computational velocities can be increased by more sampling. We observed that as spatial density of the tracks increases from levels 1 to 3, average error becomes smaller for radius ranging from 1 to 5 mm due to increment of track features. However, if the track layout becomes too compact from radius 0 to 0.5 mm, error increases due to poor feature quality.

**Figure 8:**
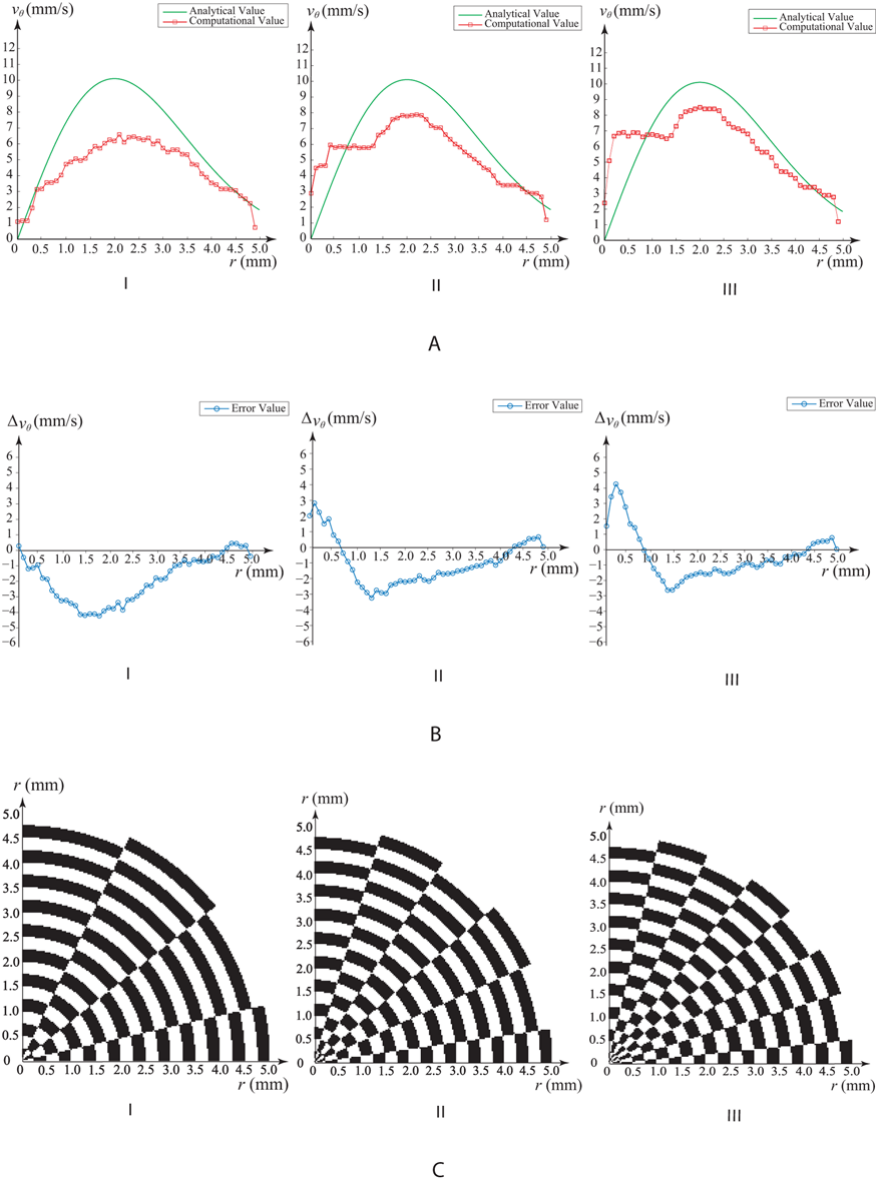
Tracking accuracy of rotation using motion estimation algorithm. Comparison of analytical and computational flow velocities in the radial direction is illustrated. Velocity profiles and their differences are shown in (a) and (b). Quadrant of the vortical grid is displayed in (c). Variation of feature density adjusts the moderation and extent of flow grid prediction.

The results demonstrated that the tracking accuracy decreases in regions towards the centre as the grid intervals become denser (Figure 8). However, where definition of the tracks becomes clearer due to better spacing, tracking improves. Increase in track quality can enhance tracking capabilities but is limited to regions away from the vortex centre. Degrading of track features quality results in higher deviation from the true flow field; nevertheless, it is able to give a more moderate variation of this error and also prevent signal aliasing in the compact grid region.

#### B.2. Variation of Noise and Smoothing

Using the same experimental setup in the previous section, we have analyzed the accuracy of profile of computational velocity decline because of the addition of noise to images used in tracking, and try to understand the balance between suppression of noise and loss of signal features due to smoothing. It may be worthwhile noting that we are also interested to examine the tracking effectiveness due to blur track features as well. For this reason, we implement the mean filter for reduction of noise by blurring the image.

**Figure 9:**
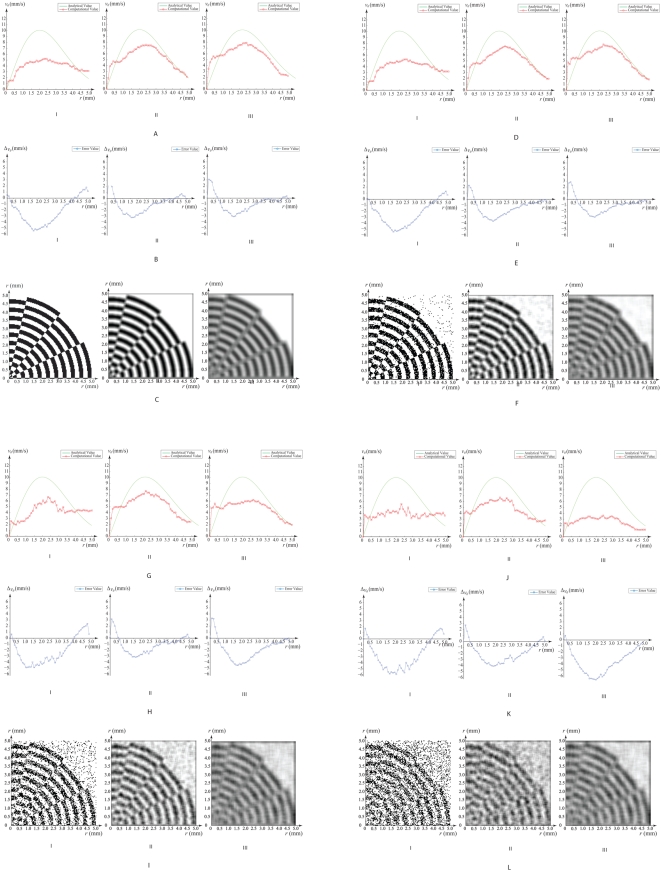
Tracking accuracy of rotation based on variation of noise in image. Comparison of analytical and computational flow velocities in the radial direction is illustrated. Velocity profiles and their differences are shown in (a,d,g,j) and (b,e,h,k) respectively. Quadrant of the vortical grid is displayed in (c,f,i,l). Variation of feature density adjusts the moderation and extent of flow grid prediction.

We have applied different smoothing filter masks that have widths at 1 by 1, 5 by 5 and 9 by 9 pixels. Gaussian noise is added to the images at percentage (%) of 0, 10, 30 and 50 incrementally. Note that the overall average error increases for radius ranging from 1 to 5 mm for every stage of additional noise input into the tracking images. However, when smoothing is applied to the images, the velocity profile improves in accuracy with respect to the analytical one as a result of the suppression of noise signals. This can be illustrated by parts (a) to (h) of Figure 9, that corresponds to velocity and error profiles derived from tracking based on 0, 10, and 30% noise to image addition. Nevertheless, we also observed that for higher noise added to images with percentages such as 30% or 50%, over smoothing using a (9×9) pixel filter mask size reduces the tracking accuracy relative to that when a (5×5) pixel smoothing mask is applied. The results describe the effect of over-smoothing after the noise addition has reached a certain threshold.

## Discussion

The tracking can be performed using intensity images to produce in-plane flow field without velocity encoding during scanning. Therefore, the overall processing time is still significantly shorter than deciphering velocity-encoded phase contrast images into similar flow field information. However, we acknowledge the offset in accuracy that is dependent on parameters such as the tracking effectiveness of the motion estimation algorithm, temporal resolution and the quality of the track features in the intensity images. This can render the computational prediction of flow to an unrealistic extent.

The performed experiments have shown that the proposed method using motion estimation of intensity images can be used to produce flow fields for qualitative analysis. We have varied a few study parameters such as the image size, sampling window size of the motion estimation algorithm, signal to noise ratio of the image and smoothing filter mask size. Further studies based on different methods of motion estimation such as block matching or affine flow models [39] can be carried out to test the tracking system. Nevertheless, in order to maintain the focus of our study, which is to understand the parameters that affect tracking, we use the pyramidal Lucas Kanade optical flow algorithm [33], [40] as the role model here. This optical flow has been effectively applied onto tracking of rigid objects with no changes of shape. This implies that it actually captures the motion of scene objects with the exclusion of expansions or contractions, as well as deformations [41].

We have proposed a method of varying spatial features within the images. Increment in the spatial dimension of the vortical track size was followed by a reduction in features and this lowered the tracking accuracy as demonstrated by our surface response error graphs. As the image or sampling window size of the optical flow algorithm increased, the error decreased and tracking can reach an optimal level. The use of analytical data enabled us to validate information for the motion estimation approach as outlined in this paper. The same principles can be tested using different track feature layout such as Cartesian grids instead of polar ones. In addition, translation of grids in addition to rotation can be studied as well. But since we are interested to examine rotation of blood using fluid motion tracking, a system validation of tracking rotational features will be more important and therefore studied here.

Although our results demonstrated that the flow prediction discrepancies are not significant, the accuracy of a computed motion estimation approach could have been limited by the quality of signal representation using the magnetic resonance images in real life. In our experiments, we had devised artificial intensity images simulating the presence of low MR-signals corresponding to incoherent proton spins in an idealized layout. The data set will not, however, reflect the organization of these signal registrations in non-continuous distribution from the MR image of a vortex. However, since our study was to verify the underlying computational algorithm of the system, it is feasible and justifiable to test the technique with a non-realistic signal registration in the simulated images at the initial stage.

**Figure 10:**
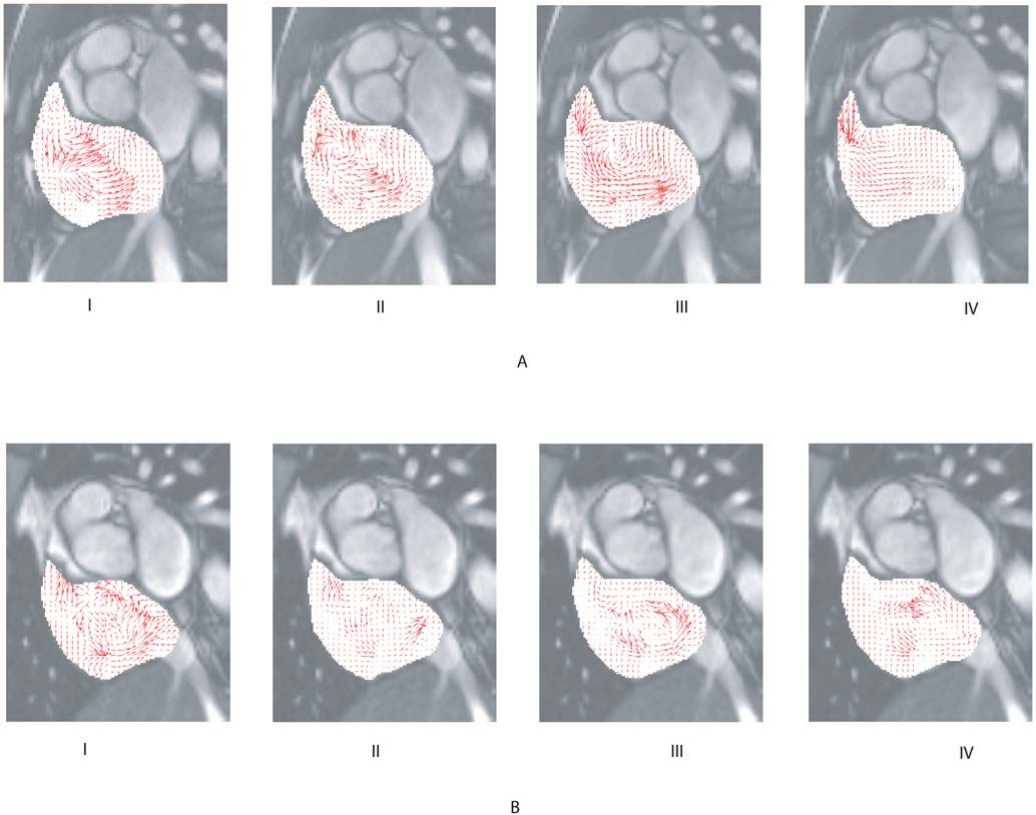
Cardiac flow results based on case study subjects. The tracking accuracy is dependent very much on the spatial and temporal resolution of the images. In addition, the existence of asynchronous proton spins must be present in order to present feature trackers for the motion estimation algorithm. Magnetic resonance (MR) fluid motion tracking can produce a quick insight into the flow behavior of blood before making decision for more detailed but time-consuming velocity-encoding scans.

Fluid motion tracking on real data such as the True FISP MR intensity images is trialled for human right atrial flow generation. Configuration of the imaging is optimally set to produce the best intensity quality pertaining to magnetic resonating blood clouds of asynchronous proton spins. Two case study subjects are chosen for this experiment. Both sets of flow results (Figure 10) revealed a dominant counter-clockwise vortex in the right atrial flow at one stage of the cardiac cycle. Sample sets of magnetic resonance images pertain to phases numbered from 10 to 13 and from 17 to 20 out of 25 phases for the first and second subject respectively can be referred to in Appendix S2. These results are based on preliminary testing of the tracking framework using a few test cases. Further validation using well-established velocity-encoded imaging modalities such as phase contrast magnetic resonance imaging can be performed to establish its reliability.

### Conclusion

We were able to provide some form of validation for a computed motion estimation approach in flow tracking and visualization. We have created artificial signal image data based on an analytical vortex. The images were simulated with an unrealistic assumption of perfect signal intensity contrast at equal geometrical intervals. However, since our main objective was to test the computational tracking mechanism of the approach, it was practical and instructive to produce idealised data sets for calibration and error estimation. Once proven reliable, the proposed system in our study will be useful for non-invasive tracking of blood flow using magnetic resonance images without modification of the standard True FISP MR image scanning.

Additionally, there may be a need to verify fluid motion estimation using experimental data generated from other imaging modalities. The same experiments can be performed using information from other flow measurement techniques as input to fluid motion tracking. For future studies, we can describe experiments that perform system verification of our methodology using flow fields derived from phase contrast magnetic resonance imaging. The whole purpose of this study is to perform a thorough performance evaluation of the suggested theory and implemented system before it can be further validated using real magnetic resonance images and well-established velocity encoded magnetic resonance imaging. We saw the need to organise information related to the system and to present the parameter dependencies in the best light possible before proceeding to the next stage.

## Supporting Information

Figure S1 Cine-magnetic resonance images of the heart. *The flow patterns of blood in the heart chambers can be traced by observation using cine-magnetic resonance images that are played at an appropriate speed to register the cardiac flow motion into our brains. We extended this activity based on the implementation of a computer vision program to perform the same ‘tracking’
(2.93 MB EPS)

Appendix S1 Definition of optical flow constraint
(0.04 MB DOC)

Appendix S2 Magnetic resonance images of case study subjects
(0.21 MB DOC)
