# Supplementary material for: Theory and Validation of Magnetic Resonance Fluid Motion Estimation Using Intensity Flow Data
Source: PLoS One. 2009 Mar 9;4(3):e4747. doi: 10.1371/journal.pone.0004747 (PMC2651647; doi:10.1371/journal.pone.0004747)
Supplement: Appendix S1 — Definition of optical flow constraint (0.04 MB DOC) [file pone.0004747.s002.doc]

## Appendix S1

**Definition of optical flow constraint**

This section highlights the basis of differential optical flow using the motion or optical flow constraint. We denote pixel intensity by *I*(*x*,*y*,*t*). Assuming spatio-temporal variation in intensity signal, we obtain

(1)

Applying a 1^st^ order Taylor series expansion, Eq. (1) becomes

(2)

whereby *ε* represents higher order terms. If the brightness of a particular point in the pattern is constant, we have

(3)

This can also be written as

(4)

Definition of $v_{x}=\frac{\delta x}{\delta t}$ and $v_{y}=\frac{\delta y}{\delta t}$ yields

(5)

Therefore, the optical flow constraint equation can be rewritten as

(6)

The optical flow vector $\mathop{v}\limits^{\to}$ has two components *v_x_* and *v_y_* describing the motion of a point feature in *x* and *y* direction with the spatial gradient of intensity is denoted by $\nabla I$. Therefore, the linearized version of the brightness constancy assumption yields the optical flow constraint given by

(7)
