# Supplementary material for: Theory and Validation of Magnetic Resonance Fluid Motion Estimation Using Intensity Flow Data
Source: PLoS One. 2009 Mar 9;4(3):e4747. doi: 10.1371/journal.pone.0004747 (PMC2651647; doi:10.1371/journal.pone.0004747)
Supplement: Appendix S2 — Magnetic resonance images of case study subjects (0.21 MB DOC) [file pone.0004747.s003.doc]

## Appendix S2

**Magnetic resonance images of case study subjects**

The images for phases 10 to 13 and from phases 17 to 20 within a cardiac cycle of 25 phases are used for magnetic resonance fluid motion tracking. The temporal images can be presented in cine-mode. With a well-coordinated visual registration of the flow patterns into our brains from the video display of these images, we can deduce the rotational motion of blood within the heart chamber heuristically.

| *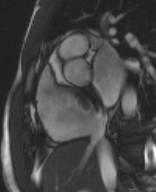* | *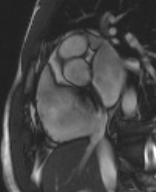* | *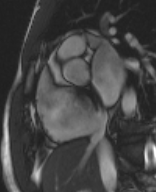* | *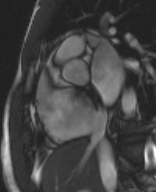* |
| --- | --- | --- | --- |
| *(i) phase 10 of 25* | *(ii) phase 11 of 25* | 1. *phase 12 of 25* | 1. *phase 13 of 25* |

(a) Case study subject 1

| *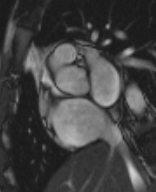* | *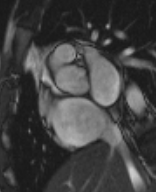* | *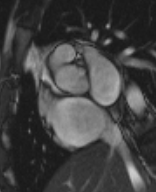* | *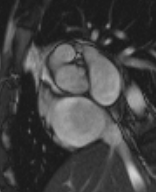* |
| --- | --- | --- | --- |
| *(i) phase 17 of 25* | *(ii) phase 18 of 25* | 1. *phase 19 of 25* | 1. *phase 20 of 25* |

(b) Case study subject 2

**Figure S2. Cine-magnetic resonance images of the heart.** The flow patterns of blood in the heart chambers can be traced by observation using cine-magnetic resonance images that are played at an appropriate speed to register the cardiac flow motion into our brains. We extended this activity based on the implementation of a computer vision program to perform the same ‘tracking’.
